# Improving the anthocyanin accumulation of hypocotyls in radish sprouts by hemin-induced NO

**DOI:** 10.1186/s12870-022-03605-w

**Published:** 2022-04-30

**Authors:** Nana Su, Ze Liu, Lu Wang, Yuanyuan Liu, Mengyang Niu, Xin Chen, Jin Cui

**Affiliations:** 1 College of Life Sciences, Nanjing Agricultural University, Nanjing, 210095 Jiangsu China; 2 School of Environmental and Life Sciences, University of Newcastle, Callaghan, NSW 2308 Australia; 3 Institute of Industrial Crops, Jiangsu Academy of Agricultural Sciences, Nanjing, 210014 Jiangsu China

**Keywords:** Hemin, Anthocyanin, Nitric oxide, Heme oxygenase-1, Radish sprouts

## Abstract

**Background:**

The health benefits of anthocyanins impel researchers and food producers to explorer new methods to increase anthocyanin contents in plant foods. Our previous studies revealed a positive role of nitric oxide (NO) in anthocyanin accumulation in radish (*Raphanus sativus* L.) sprouts. The application of hemin, an inducer of heme oxygenase-1 (HO-1), can effectively elevate NO production in vivo. Hemin treatment also improves plant growth and stress tolerance. This study is aimed to assess the effects of hemin treatment on anthocyanin production in radish sprouts, and to investigate whether NO signalling is involved in this process.

**Results:**

The application of hemin significantly up regulated the expressions of many anthocyanins biosynthesis related structure and regulatory genes, leading to increased anthocyanins accumulation in radish hypocotyls. Hemin treatment also raised NO contents in radish sprouts, probably through enhancing nitrate reductase (NR) activity and *Nitric Oxide-Associated 1* (*NOA1)* expression. Comparing the effects of Zinc Protoporphyrin (ZnPP, HO-1 activity inhibitor), Sodium Nitroprusside (SNP, NO donor) and carboxy-PTIO (cPTIO, NO-scavenger) on anthocyanin and NO production, a positive role of NO signalling has been revealed in hemin-derived anthocyanin accumulation. A positive feedback loop between HO-1 and NO may be involved in regulating this process.

**Conclusions:**

Hemin induced anthocyanin accumulation in radish sprouts through HO-1 and NO signalling network.

**Supplementary Information:**

The online version contains supplementary material available at 10.1186/s12870-022-03605-w.

## Background

Anthocyanins are a type of flavonoid pigments found in many plant foods, providing the red, orange, violet and blue colours for flowers, fruits, and leaves [1]. Plants produce anthocyanins as a protective mechanism against environmental stressors (ultraviolet light, cold temperatures, and drought) [2, 3] and biotic attack (herbivory and pathogen) [4, 5]. Moreover, flowers accumulate anthocyanins to attract pollinators and seed dispersers [6–8]. In addition to the roles in plants growth and development, anthocyanin have been demonstrated with many pharmacological benefits to human health, such as antioxidant, anti-inflammatory, preventing age-related cardiovascular disease and neurodegenerative disease [9, 10]. Therefore, increasing anthocyanin concentration in fruit and vegetable could potentially promote human health.

The anthocyanin biosynthesis pathway is an extension of the general flavonoid pathway. Phenylalanine ammonia lyase (*PAL*), chalcone synthase (*CHS*), chalcone isomerase (*CHI*) and flavanone-3-hydroxylase (*F3H*) are the key genes related to the general phenylpropanoid pathway. Therefore, these genes are involved in the biosynthesis of all downstream flavonoids, and generally be classified as early biosynthesis genes (EBGs). While late biosynthesis genes (LBGs) are specifically required for the biosynthesis of anthocyanins, such as dihydroflavonol 4-reductase (*DFR*), anthocyanidin synthase (*ANS*), leucoanthocyanidin dioxygenase (*LDOX*) and UDPglucose:flavonoid-3-O- glucosyltransferase (*UF3GT*) [11]. The expressions of these anthocyanin structural genes are conservatively controlled by the MYB-bHLH-WDR (MBW) transcription factor complex [12, 13]. Two R2R3-MYB genes, *Production of anthocyanin pigment1* (*PAP1/MYB75, At1g56650*) and *PAP2* (*MYB90*, *At1g66390*), has been identified in *Arabidopsis* as positive regulators for the expression of anthocyanin biosynthesis genes [14–16]. The *PAP1* homologs in snapdragon, radish and cotton have also been revealed stimulating anthocyanin production [17–19].

Previous study in our group has revealed 0.5 mM sodium nitroprusside (SNP, a NO-releasing compound) application significant increased anthocyanin accumulation in radish sprout, along with enhanced endogenous NO levels [18]. Interestingly, an opposite phenotype has been reported in Lycium fruits during ripening, as the SNP supply and endogenous NO content negatively correlated with anthocyanin biosynthesis [20]. To further clarify the role of NO in anthocyanin production, we used hemin treatment in this study to induce the endogenous NO content. The effectiveness of hemin in trigger endogenous NO levels has been evidenced previously in cucumber and tomato lateral roots [21, 22]. As a heme oxygenase-1 (HO-1) inducer, hemin application could improves plant stress tolerance in different plant species under various abiotic stress conditions [23–25]. Thus, hemin treatment has the potential to benefit plant growth, rather than to generate stress induced anthocyanin production.

We used cherry radish in the present study, as it is a nutritious and popular vegetable worldwide [26]. Moreover, with the red hypocotyls resulting from anthocyanin accumulation, radish sprouts could provide visual evidence for the biosynthesis of anthocyanins. Our results here suggested hemin could induce anthocyanin accumulation in plants through NO signalling pathway.

## Results

### Effects of hemin application on anthocyanin accumulation and endogenous NO production in radish sprouts

**Fig. 1:**
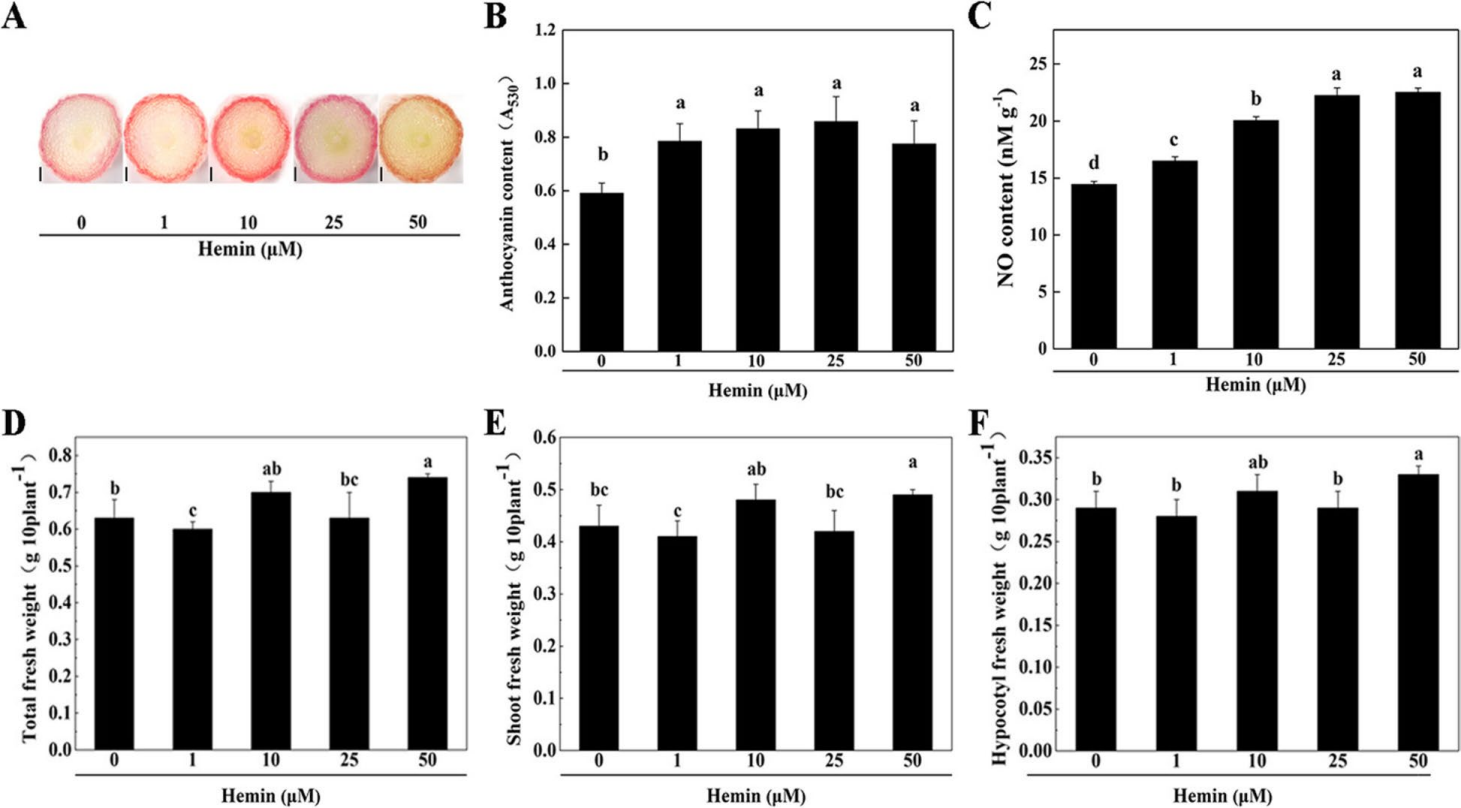
Effects of hemin treatments on anthocyanin accumulation, NO production and fresh weight in radish sprouts. **A** The hypocotyl cross sections of radish sprout cultured with 0, 1, 10, 25 and 50 μM hemin supplements. Bar = 0.5 mm. **B-C** Anthocyanin content **(B)** and NO content **(C)** in radish hypocotyls after 0, 1, 10, 25 and 50 μM hemin treatments. **D-F** Total fresh weight **(D)**, shoot fresh weight **(E)** and hypocotyl fresh weight **(F)** of radish sprouts after 0, 1, 10, 25 and 50 μM hemin treatments. The measurement resolution of the image was 3072*2304. The values were means ± standard deviation (SD) of the three independent experiments with at least three replicates for each. Different letters indicate significant differences among treatments (*P* < 0.05)

The anthocyanin contents in the hypocotyls of radish sprouts were examined after cultured with 1, 10, 25 and 50 μM hemin for 48 h. As shown in Fig. 1, significantly higher anthocyanin contents were observed in all hemin treated hypocotyls, along with enhanced NO levels, as compare to no-hemin control. However, the increase of anthocyanin was disproportionate to the raise of NO amount. Clearly, the anthocyanin biosynthesis in radish hypocotyl was highly sensitive to low level of hemin application, but quickly plateaued out when supplied with higher concentrations of hemin concentrations (Fig. 1B). In contrast, the endogenous NO contents exhibited a roughly linear increase by hemin applications up to 25 μM (Fig. 1C). The application of 50 μM hemin increased radish total fresh weight, shoot fresh weight and hypocotyl fresh weight (Fig. 1D-F).

**Fig. 2:**
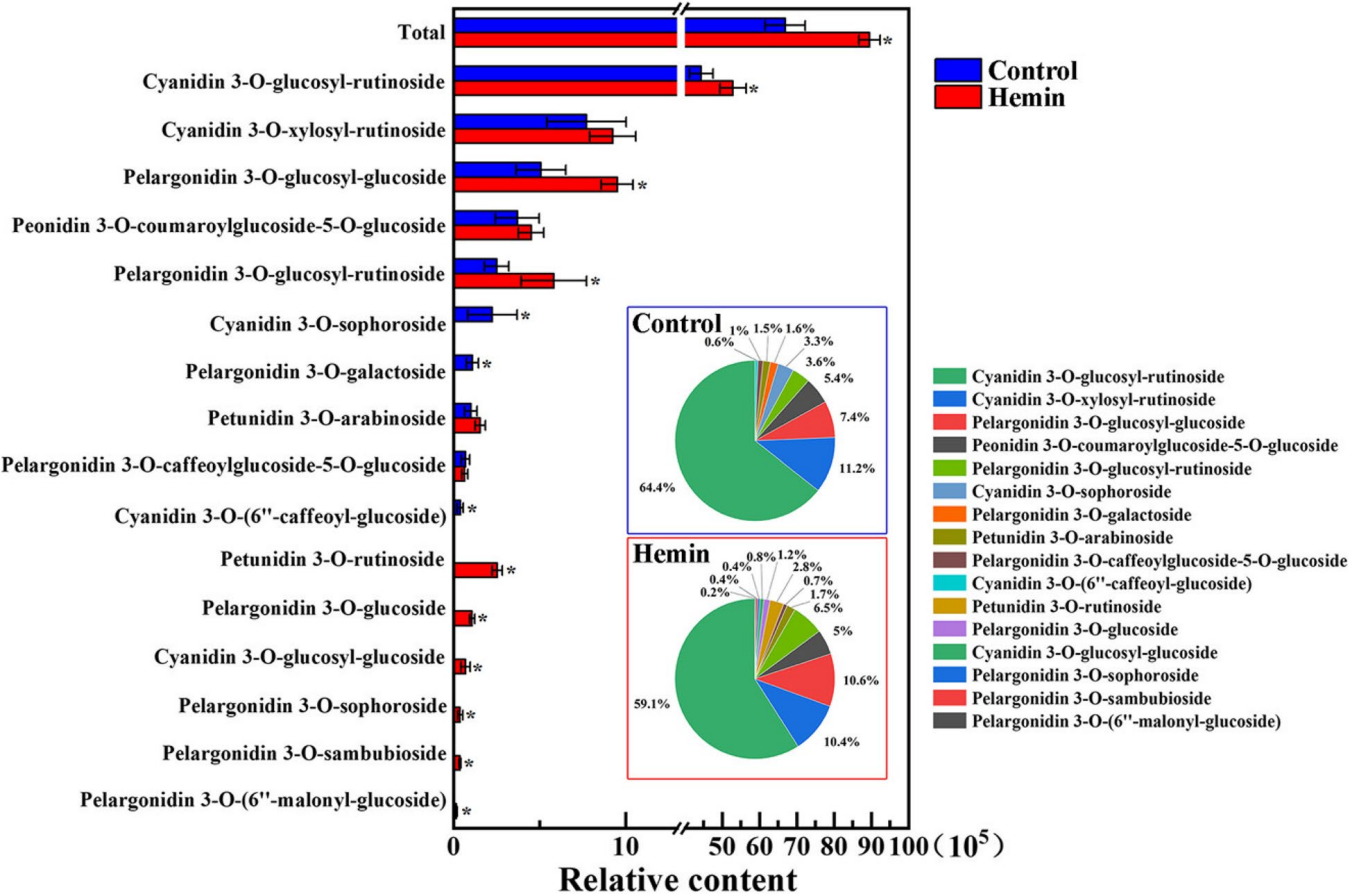
Effects of hemin treatment on the contents and proportions of anthocyanin monomers. The relative contents of anthocyanin monomers were shown as mean ± standard deviation (SD) of three independent experiments with at least three replicates for each. Asterisk indicates significant difference between control (no hemin) and hemin (10 μM) treatments (*P* < 0.05)

The effects of hemin treatment on the contents of different anthocyanin monomers were also assessed using liquid chromatography–mass spectrometry (LC–MS). Among the different anthocyanin monomers, cyanidin 3-O-glucosyl-rutinoside was the main component in radish hypocotyls, which accounted for 64.4% of the total anthocyanin, followed by cyanidin 3-O-xylosyl-rutinoside (11.2%), pelargonidin 3-O-glucosyl-glucoside (7.4%), peonidin 3-O-coumaroylglucoside-5-O-glucoside (5.4%) and pelargonidin 3-O-glucosyl-rutinoside (3.6%), and others only made up to less than 8% of the total anthocyanin contents (Fig. 2). 10 μM hemin treatment exhibited a ~ 30% higher total anthocyanin level, mainly attributed to the 19.1%, 87.4%, 21.5% and 132.8% increases of cyanidin 3-O-glucosyl-rutinoside, pelargonidin 3-O-glucosyl-glucoside, pelargonidin 3-O-glucosyl-rutinoside and petunidin 3-O-rutinoside respectively (Fig. 2). In contrast, the amounts of cyanidin 3-O-sophoroside, pelargonidin 3-O-galactoside, cyanidin 3-O-(6’’-caffeoyl-glucoside) were significantly reduced in hemin cultured radish hypocotyls (Fig. 2). The adjusted monomer proportions probably led to the slight colour changes of the hypocotyl cross sections shown in Fig. 1A.

### Effects of NO on anthocyanin contents in hypocotyls of radish sprouts

**Fig. 3:**
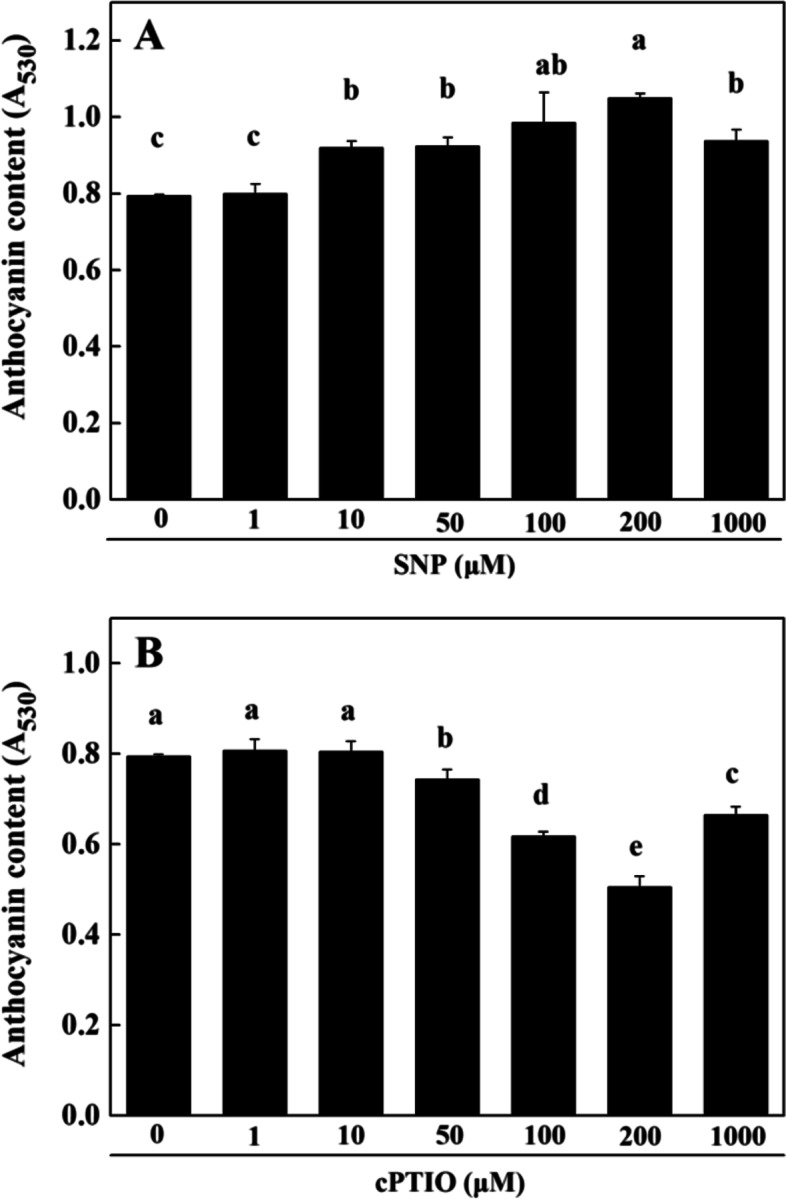
Radish hypocotyl anthocyanin contents in response to SNP (**A**) and cPTIO (**B**) application. Total anthocyanin contents were represented as means ± standard deviation (SD) of the three independent experiments with at least three replicates for each. Different letters indicate significant differences among treatments (*P* < 0.05)

To further investigate the effect of NO on anthocyanin accumulation in the radish hypocotyls, Sodium Nitroprusside (SNP, an exogenous NO donor) and carboxy-PTIO (cPTIO, a specific NO-scavenger) were then used. As shown in Fig. 3A, 10, 50, 100, 200 and 1000 μM SNP applications significantly enhanced anthocyanin contents, with the highest amount achieved by 200 μM SNP treatment. On the other hand, 50, 100, 200 and 1000 μM cPTIO treatments significantly decreased anthocyanin contents in radish hypocotyls, with the lowest anthocyanin level detected by 200 μM cPTIO treatment (Fig. 3B). Taken together, anthocyanin accumulation in hypocotyls positively correlated with NO production at low to medium levels, whereas high level NO exposure could suppress anthocyanin accumulation.

### The relationship of nitric oxide and hemin in anthocyanin induction

**Fig. 4:**
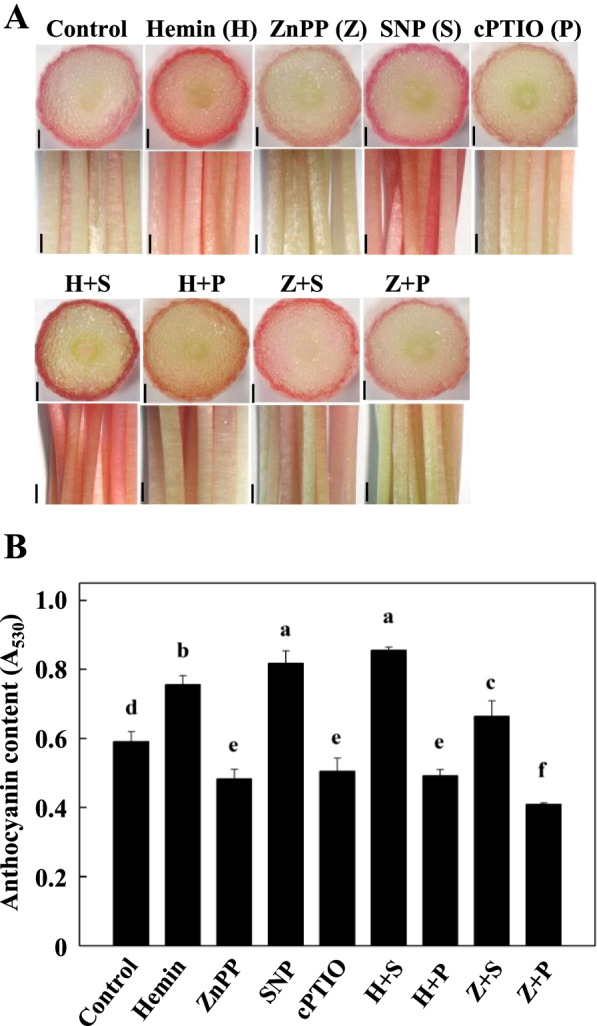
Effects of hemim, ZnPP, SNP, cPTIO and their combinations on anthocyanin accumulation in radish hypocotyls. **A** Representative images of the hypocotyl cross sections (top) and the hypocotyls (bottom), bars = 0.5 mm in cross section, and 1.0 mm in hypocotyls. **B** The anthocyanin contents in radish hypocotyls under different treatments. The measurement resolution of the image was 3072*2304. Data represented in mean ± standard deviation (SD) of the three independent experiments with at least three replicates for each. Different letters indicate significant differences among treatments (*P* < 0.05)

The biological effects of hemin application have been largely attributed to its induction of HO-1 *in vivo* [27, 28]. To test if the role of hemin in anthocyanin accumulation was related to NO production, Zinc (II) Protoporphyrin IX (ZnPP) was used, due to its specific role in HO-1 activity inhibition. ZnPP has been well documented in medical research able to reverse the effects of hemin. In human monocytes, Hemin inhibits apoptosis, while ZNPP reverses this effect. Hemin also promoted the formation of adventitious roots in cucumber explants in a dose-dependent manner, while treatment with Znpp resulted in a significant reduction in hemin-induced adventitious roots [29–31]. Compare to control, ZnPP treatment significantly decreased anthocyanin accumulation (Fig. 4), indicated a positive correlation between HO-1 activity and anthocyanin content. If hemin increase anthocyanin content through promoting NO production, we may expect the suppression of anthocyanin content by ZnPP could be abolished by the SNP application. Indeed, ZnPP + SNP treatment significantly increased anthocyanin levels in radish hypocotyls (Fig. 4). Interestingly, the co-application of ZnPP and ePTIO led to an even lower level of anthocyanin, as compare to single chemical treatments (Fig. 4), implying hemin and NO may act addictively in anthocyanin accumulation in radish hypocotyls.

### Expression of anthocyanin-related biosynthesis structural and regulatory genes under different treatments

To explore the molecular basis of the hemin and NO in anthocyanin accumulation, we then tested the expression levels of anthocyanin biosynthesis-related structural and regulatory genes in the hypocotyls of radish sprouts. 8 families of anthocyanin biosynthesis genes were identified from radish hypocotyl transcriptome data (obtained in our previous studies). The members and their expression levels were listed in Supplemental Table 1. For *F3H*, *DFR*, *ANS*, *LDOX* and *UFGT*, the gene with the highest FPKM value in each family was chose for further expression. For *PAL*, *CHI* and *CHS*, several genes shown relatively high FPKM values. The transcription levels of the top three members in each family were then verified by RT-qPCR (Supplemental Fig. 1), and the dominant genes were chosen for further experiment in response to different chemical treatments.

**Fig. 5:**
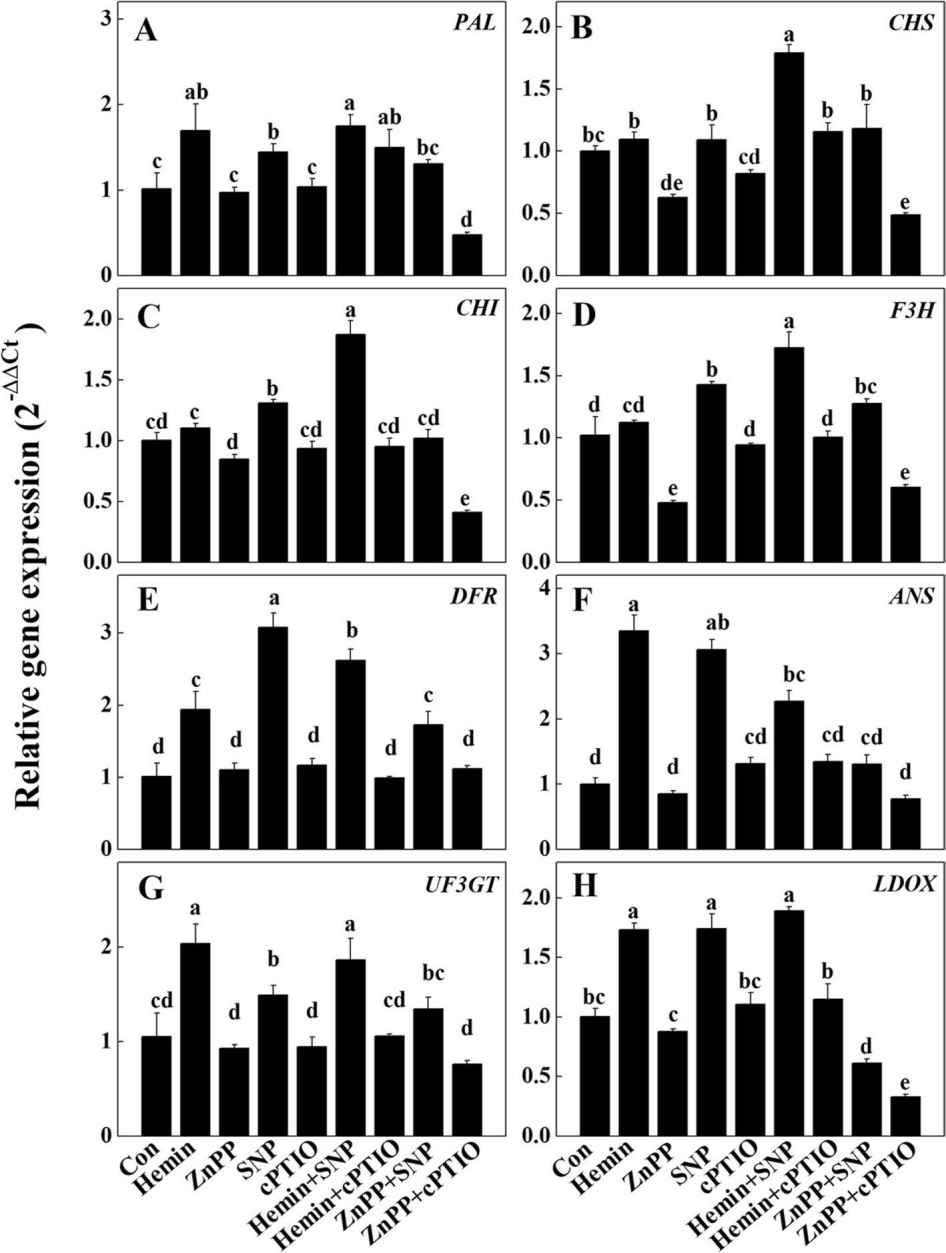
Effects of hemin, ZnPP, SNP and cPTIO on the expression of anthocyanin biosynthesis-related structural genes. The expression level of **A** *PAL*, **B** *CHS*, **C** *CHI*, **D** *F3H*, **E** *DFR*, **F** *ANS*, **G** *UF3GT* and **H** *LDOX*. Each gene here was the dormant member in corresponding gene family. Expression levels were represented in means ± SD of three independent experiments with at least three replicates for each. Different letters indicate significant differences among treatments (*P* < 0.05)

As shown in Fig. 5, *PAL* is the only EBG gene stimulated by hemin supply; ZnPP treatment reduced *CHS* and *F3H* transcription; SNP application increased the expressions of three EBGs *PAL*, *CHI* and *F3H*; while the transcripts of all four tested EBGs remained similar abundances after cPTIO treatment. In contrast, hemin and SNP treatments significantly up regulated the transcriptions of all tested anthocyanin LBGs (*DFR*, *ANS*, *UF3GT*, *LGOX*); whereas ZnPP and cPTIO treatments had no effects on these LBGs expression levels in radish hypotocyls (Fig. 5). Moreover, hemin + SNP significantly enhanced the transcriptions of all eight tested anthocyanin biosynthesis-related structure genes; ZnPP + cPTIO suppressed the expressions of EBGs and *LDOX* (Fig. 5). Notably, hemin + cPTIO successfully abolished the activation of LBG transcriptions by hemin, indicating hemin may act through NO signalling in regulating these anthocyanin biosynthesis genes; while SNP + ZnPP treatment decreased LBG expressions as compare to SNP-only, suggesting an opposite signalling pathway as HO-1 activity may be required for NO to exert its role (Fig. 5). Together, these results implied a crosstalk between HO-1 and NO signalling in regulating anthocyanin biosynthesis.

**Fig. 6:**
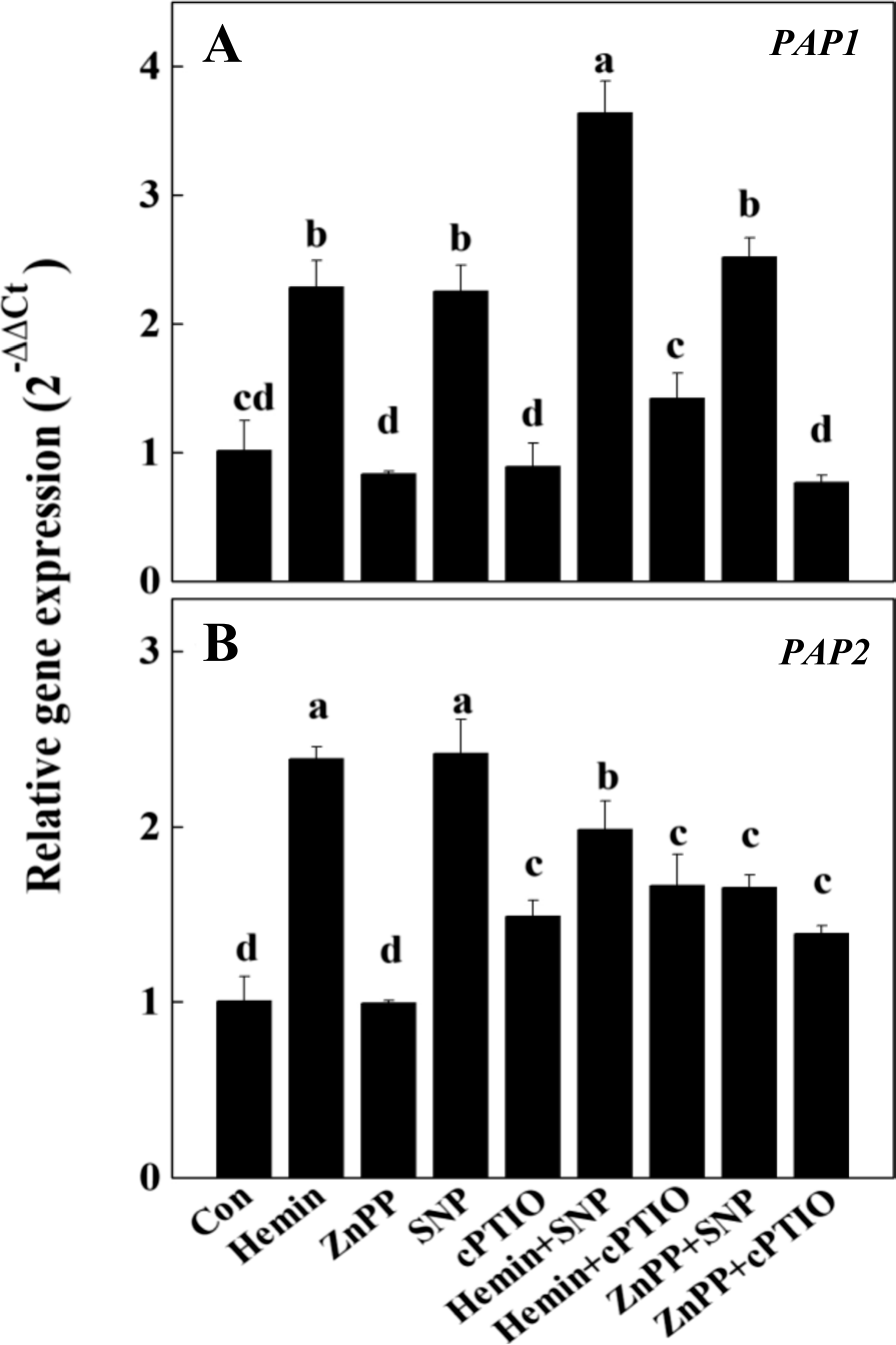
Effects of hemin, ZnPP, SNP and cPTIO on the expressions of *PAP1* (**A**) and *PAP2* (**B**). Expression levels were represented in means ± SD of three independent experiments with at least three replicates for each. Different letters indicate significant differences among treatments (*P* < 0.05)

In addition, hemin and SNP treatments greatly stimulated the transcriptional levels of two radish *PAP* homologs, *PAP1* and *PAP2*, in radish hypocotyls (Fig. 6). Noteworthily, the hemin + SNP treatment gave rise to a dramatic raise of *PAP1* expression (3.6 folds of that in control). Compare with the 128% and 124% increasements of *PAP1* transcripts by hemin-only and SNP-only respectively, these two chemicals may act synergistic in stimulating *PAP1*expression, and thereby the biosynthesis of anthocyanin. However, hemin + SNP application resulted less *PAP2* transcripts than hemin-only or SNP-only treatments, implying a different regulatory pattern of hemin and NO signalling in modifying *PAP2* expression.

### NO content, NO biosynthesis-related enzymes activities and gene expression under various chemical treatments

**Fig. 7:**
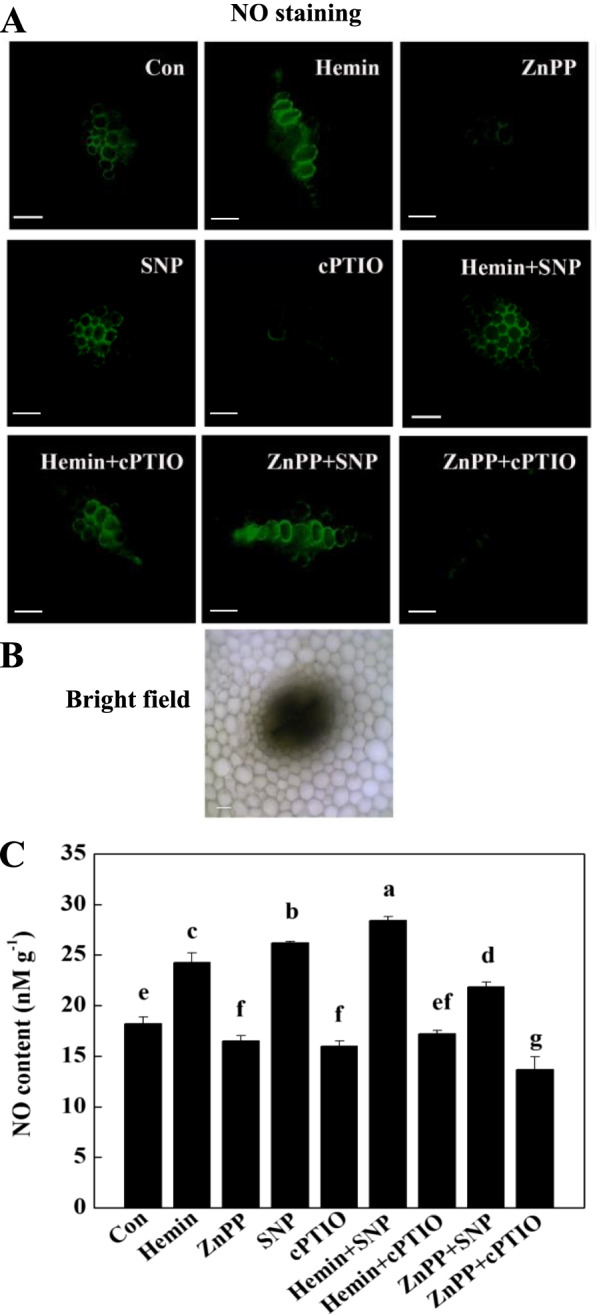
The NO contents in the radish hypocotyls in response to different treatments. **A** Representative fluorometric images of the radish hypocotyl cross sections stained by NO specific tracer DAF FM-DA after according chemical treatments. Bar = 50 μm. **B** A representative bright field image of the radish hypocotyl cross section. **C** The NO contents in the hypocotyls of radish sprouts measured by nitrate reductase method. The measurement resolution of the image was 1388*1044. The values were means ± standard deviation (SD) of the three independent experiments with at least three replicates for each. Different letters indicate significant differences among treatments (*P* < 0.05)

To further investigate the relationship between hemin and endogenous NO production, we tested the NO contents in response to the chemical treatments as above. Using the specific NO fluorescent probe, 4-Amino-5-methylamino- 2’,7’-difluorofluorescein diacetate (DAF-FM DA), NO signals were detected mainly in the stele tissues of radish hypocotyls (Fig. 7A). Compare to control, hemin, SNP, hemin + SNP, ZnPP + SNP treatments significantly increased NO contents, whereas ZnPP, cPTIO and ZnPP + cPTIO suppressed the NO levels in radish hypocotyls (Fig. 7C). Interestingly, ZnPP + SNP treatment caused a small induction of NO in compare to control, but still significantly lower than that of SNP-only; moreover, hemin + SNP generated higher NO level than hemin or SNP only (Fig. 7C). Hence, consistently, HO-1 activity was probably involved in SNP-derived NO production.

**Fig. 8:**
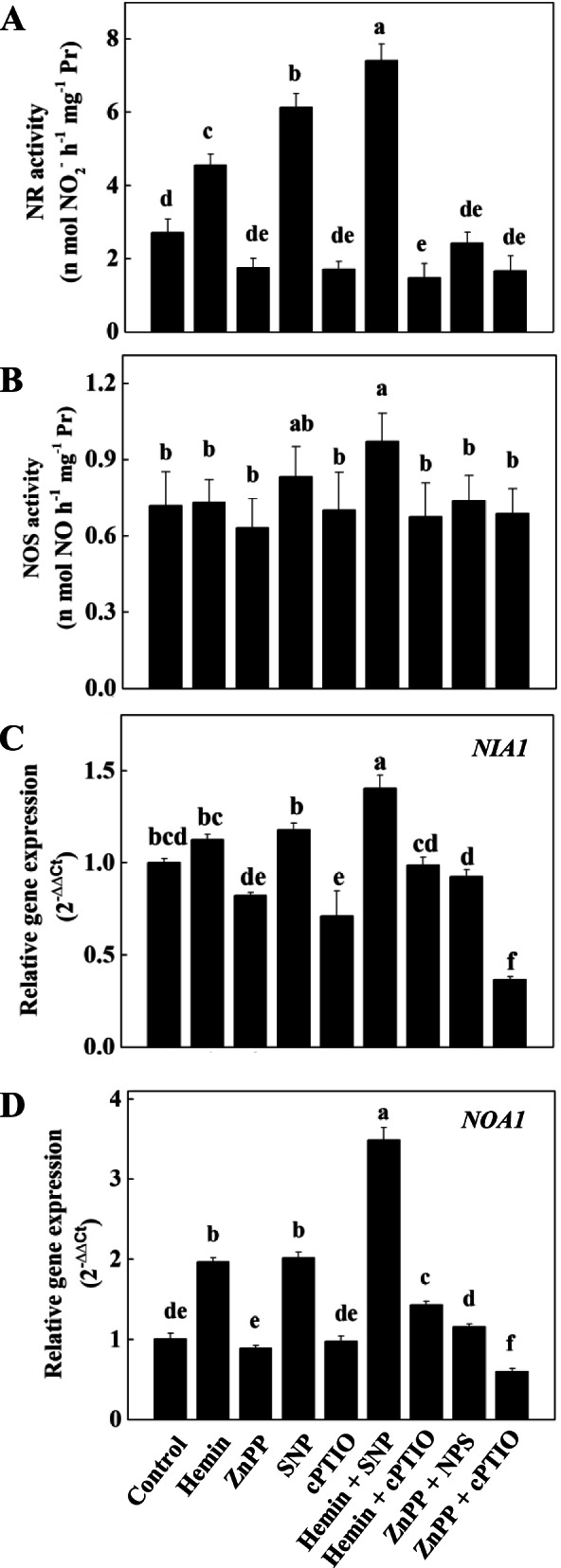
Effects of different treatments on the activities of key NO biosynthesis enzymes and encoding genes. **A** and **B** The enzyme activities of NR (**A**) and NOS (**B**) in radish hypocotyls. **C** and **D** The relative expression levels of NIA1 (**C**) and NOA1 (**D**) in radish hypocotyls. RsActin was used as internal control. The values were means ± standard deviation (SD) of the three independent experiments with at least three replicates for each. Different letters indicate significant differences among treatments (*P* < 0.05)

Subsequently, we measured the activities of two key enzymes as well as the expression levels of NO biosynthesis-associated genes. Nitrate reductase (NR) reduces nitrate to nitrite using electrons from NAD(P)H, and also lead to NO production from nitrite due to its nitrite reductase activity [32, 33]. While nitric oxide synthases (NOS) catalyzing the production of nitric oxide (NO) from L-arginine. As shown in Fig. 8A, hemin, SNP and hemin + SNP cultured radish hypocotyls presented higher NR activities than control treatment. However, the activity of NOS was only slightly increased in hemin + SNP treatment (Fig. 8B). In addition, higher abundance of *NIA1* (Nitrate reductase [NADH]) transcripts were detected by SNP supplication, while hemin and SNP promoted the expression levels of *NOA1* (Nitric oxide associated factor; a putative *NOA1* homolog gene identified in radish transcriptome according to the Arabidopsis *NOA1* gene sequence) in radish hypotocyls (Fig. 7C). Together, the activities of key enzymes and the transcription levels of the related genes encoding were roughly correlated with the endogenous NO levels in the different chemical treatments, confirmed the positive role of hemin and SNP in NO production.

## Discussion

The purpose of this study is to test the effects of hemin application in anthocyanin induction, and to investigate whether NO signalling is involved in this process. As expected, hemin treatment enhanced anthocyanin accumulation in the hypocotyls of radish sprouts (Figs. 1 and 4). At transcriptional level, hemin application promoted the expressions of anthocyanin biosynthesis structure genes (especially the LBGs, Fig. 5), as well as two R2R3-MYB transcription factors (*RsPAP1* and *RsPAP2*, Fig. 6), which probably act as positive regulatory in anthocyanin biosynthesis same as their Arabidopsis homologs. Notably, the anthocyanin level was significantly increased by low level hemin application (33% higher anthocyanin content at 1 μM hemin as compare to no hemin control), however, further increasing hemin concentrations to 10 μM and 25 μM had much smaller effects on anthocyanin accumulation (Fig. 1). The induction of anthocyanins in vegetative tissues is often considered to be a response of plants to biotic or abiotic stress conditions [34, 35]. It is reasonable that the accumulation of anthocyanins in these tissues should be tightly regulated, to avoid unnecessary anthocyanin production due to pleiotropy, meanwhile to maintain some degree of freedom [36]. In this study, the radish seedlings were grown under optimal conditions, and the addition of hemin in the culture system had no negative effect on seedling growth. It makes sense that the increase of anthocyanin by hemin treatments was retained at reasonable levels. Hence, for health benefits, the anthocyanin contents in radish sprouts could be elevate to a certain level by hemin treatment, although a further boost would be challenging. In addition, the proportions of anthocyanin monomers were changed after hemin treatments (Fig. 2). Future studies to identify the specific biosynthesis and regulatory pathways of desired monomers would be useful for bioengineering purpose.

Besides anthocyanin, hemin applications also increased the endogenous NO levels in radish hypocotyls (Figs. 1 and 6). As a well-known HO-1 inducer, hemin induces NO production through HO-1/CO signal transduction [37]. Hemin-induced NO production has been documented in various biological processes, such as wheat endosperm development [38], tomato, rice and cucumber rooting process [22, 37, 39] and sunflower seedling growth in response to salt stress [40]. To explore the involvement of this hemin-induced NO production in anthocyanin accumulation, we compared the effects of SNP (NO donors), cPTIO (NO scavenger) and ZnPP (HO-1 inhibitor) along with hemin treatment. SNP and cPTIO applications positively and negatively regulated anthocyanin accumulation in radish hypocotyls (Figs. 3, 4, 5 and 6). Supply cPTIO together with hemin (hemin + cPTIO) significantly reduced anthocyanin and NO productions, as compare to those in hemin-only treatment (Figs. 3 and 7), confirmed the contribution of NO in hemin-derived anthocyanin accumulation. Thus, hemin (HO-1 inducer) probably increases anthocyanin production via HO-1/CO–NO signalling crosstalk. Of course, more experiments are required to confirm the increase of in vivo HO-1 activity by hemin feeding.

Interestingly, ZnPP displayed an inhibitory role in SNP-generated NO production and anthocyanin accumulation (Figs. 4, 7 and 8), suggesting an involvement of HO-1 activity in NO biosynthesis in vivo. One possible explanation is that NO may generate a signal to positively regulate HO-1 production, while restricting HO-1 activity blocks the positive feed-back loop, and therefore reduced NO and anthocyanin productions. NO-mediated HO-1 induction has been reported in many medical research studies, such as in vascular smooth muscle cell function [41] and in regulating murine macrophage-like cell line J774.1/JA-4 [42]. Obviously, future studies are required to test this hypothesis in radish sprouts and other plant systems.

By enhancing the HO-1 activity, hemin promotes cellular heme degradation to bliverdin (BV), carbon monoxide (CO) and ferrous iron (Fe^2+^) It would be interesting to test whether these endogenous products of hemin, BV, Fe^2+^ and CO also involved in this hemin-induced anthocyanin accumulation in the future studies.

## Conclusion

**Fig. 9:**
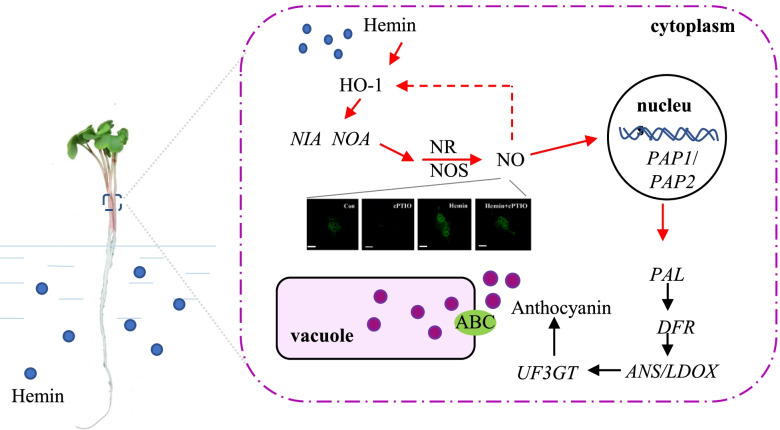
A model of how hemin induce anthocyanin accumulation in the hypocotyls of radish sprouts. Hemin probably act as a HO-1 inducer to increase NIA and NOA1 gene expression and enhance NR and NOS activities, and thereby promotes NO production. NO may induce HO-1 heme oxygenase-1 gene expression (as revealed in human muscle cell line and murine macrophage-like cell line) to form a positive feedback loop with HO-1. NO signalling probably up regulates the expression of transcription factors (PAP1 and PAP2), which could activate the transcriptions of anthocyanin biosynthesis related structural genes, and lead to anthocyanin accumulation. The measurement resolution of the image was 1388*1044. Bar = 50 μm

Taken together, this study suggested hemin application could act through NO signalling in stimulating anthocyanin accumulation in the hypocotyls of radish sprout. The possible molecular mechanism is proposed as Fig. 9. Hemin, as a HO-1 inducer, could up regulate the expression of *NIA1* and *NOA1* gene, enhance NR and NOS activities, and thereby promote NO production. NO may induce HO-1 gene expression to form a positive feedback loop with HO-1. NO signalling also stimulates the expressions of anthocyanin biosynthesis related regulatory R2-R3 MYB transcription factors (*PAP1* and *PAP2*) and structural genes (*PAL*, *DFR*, *ANS*, *LDOX* and *UF3GT*), leading to higher anthocyanin accumulation.

## Materials and methods

### Plant material and growth conditions

*Raphanus Sativus* (L.) cv. Yanghua seeds were purchased in Nanjing Wanbang Seed Industry Co., Ltd. The seeds were soaked in distilled water for 8 h and placed on moist gauze for germination for 24 h in dark at 25 ± 2℃. Uniform-sized seedlings were transferred into petri dish with two layers of moist filter paper, grown for 36 h in the dark. Seedlings were then cultured with according chemical solutions or deionized water (as control) in a growth incubator (Ningbo Haishu Safe Instrument Experimental Factory, Zhejiang, China) under LED white light (red ratio 14.1%, green ratio 81.3%, blue ratio 4.6%) for 48 h (50 ± 5 μmol·m^−2^·s^−1^ light intensity,80% relative humidity, 25 ± 2℃). The chromaticity parameters were measured by Spic-200 spectral color illuminance meter. Hemin, ZnPP, SNP and cPTIO were purchased from Sigma-Aldrich (China). For control set, 5 mL deionized water or corresponding chemicals was added into the petri dish every 12 h. The hypocotyls of the seedlings were then harvested for measurement in different experiments.

### Observation of hypocotyl cross section of Radish sprout

Radish hypocotyls were hand sectioned. The hypocotyl cross sections were visualized under a stereomicroscope (Model Stemi 2000-C; Carl Zeiss, Germany), and photographed using a digital camera (Powershot A620, Canon Photo Film, Japan, The measured resolution was 3072*2304).

### Anthocyanin content measurement

Total anthocyanin content was measured according to [43] with modification. 0.5 g fresh hypocotyls were immersed in 1% methanol hydrochloride for 24 h in the dark. The extraction solvents were then centrifuged at 5,000 g for 10 min at 4℃. The supernatants were measured by a spectrophotometry (UV-5200 spectrophotometer; Shanghai Metash Instruments Co., Ltd, China). The absorbances at 530 nm and 657 nm were used for anthocyanin content determination, using the following formular: Anthocyanin content (U·g^−1^FW) = (A_530_-A_657_ × 0.25) /Fresh weigh.

### Determination of anthocyanin monomer contents

The contents of anthocyanin monomers were determined using UPLC-MS according to [44]. 2 g fresh hypocotyl samples were grounded in liquid nitrogen and put in a 10 mL centrifuge tube containing 5 mL 0.1% acetic acid–methanol (V/V) solution for 12 h in dakness. Then the tubes with mixture were centrifuged at 14,500 g at 4℃ for 10 min, and 5 mL of supernatant was taken out and last evaporated to dryness using a rotary evaporator (LNG-T120, Taicang Hualida Laboratory Equipment Co., Ltd, Taichang, China). Add 200 μL 80% methyl alcohol (V/V) into the tubes to dissolve the dryness and then filtered with 0.22 μm regenerated cellulose filter before LC–MS/MS measurement.

The samples were analyzed by LC–MS system (G2-XS QTof, Waters). 2 μL solution was injected into the UPLC column (2.1 × 100 mm ACQUITY UPLC BEH C18 column containing 1.7 μm particles) with a flow rate of 0.4 mL/min. Buffer A consisted of 0.1% formic acid in water, and buffer B consisted of 0.1% formic acid in acetonitrile. The gradient was 5% Buffer B for 1 min, 5–95% Buffer B for 11 min, 95% Buffer B for 2 min. Mass spectrometry was performed using electrospray source in positive ion mode with MS acquisition mode, with a selected mass range of 50–1200 m/z. The lock mass option was enabled using leucine-enkephalin (m/z 556.2771) for recalibration. The ionisation parameters were the following: capillary voltage was 3.0 kV, cone voltage was 30 V, source temperature was 120 °C, and desolvation gas temperature was 400 °C. Collision energy was 20–40 eV. Data acquisition and processing were performed using Masslynx 4.1. Extraction of centroid spectra peaks with a width of 0.01 Da was used to determine the extracted ion chromatograms (EICs) from the total ion chromatogram (TIC). The data were processed by Xcalibur software, and the content of anthocyanin monomer was represented by the peak area of the sample.

### NO quantification

Radish hypocotyls (0.5 g) were averagely homogenized with 4 mL of 40 mM HEPES buffer (pH 7.2), and then the mixture was centrifuged for 10 min at 8,000 g at 4 ℃. The supernatant was gathered and tested in an A012 Nitric Oxide (NO) assay kit (Nitrate reductase method) (Nanjing Jiancheng Bioengineering Institute, Jiangsu, China). Fluorescent tracer DAF-FM-DA (3-Amino,4-aminomethy1-2’, 7’difluorescein, diacetate) were used to quantify the NO levels in radish sprout hypocotyls. The hypocotyls were hand sectioned to ~ 1 mm thickness, washed with PBS buffer (PH7.4) for three times, and then incubated with 5 μM DAF-FM DA in dark for 20 min. The samples were then washed with PBS (PH7.4) three times (10 min each time) to fully remove the surface dye. Zess Imager M2 fluorescence microscope was used for observation and photographing (The measured resolution was 1388*1044). Fluroescent signals were detected using the excitation wavelength of 495 nm, and the emission wavelength of 515 nm. The relative fluorescence intensity was calculated by Axio Vison Rel.4.8.

### Determination of NR and NOS activities

The enzyme activity of NR and NOS was detected by nitrate reductase assay kit and total nitric oxide synthase assay kit (purchased from Nanjing Jiancheng Institute of Biological Engineering). Before detecting the NR enzyme activity, the cleaned hypocotyls were soaked in the induction solution for 2 h, and then dried with filter paper and stored at -20℃ for 30 min. The total protein was extracted according to the [45]. The NR and NOS activities according to the instruction manual for determination.

### RNA isolation and qRT-PCR

Freshly harvested radish hypocotyls (100 mg) were adequately ground to powder with liquid nitrogen for RNA extraction. Total RNA was extracted using the Trizol reagent (Invitrogen, Gaithersburg, MD) following manufacturer’s instruction, and finally dissolved in 50 μl RNase-free water. The extracted RNA was disposed with DNase I (RNase-free, Transgen®) at 25 °C for 30 min, followed by performance of reverse transcription according to the manufacturer’s instruction (TransScript® First-Strand cDNA Synthesis SuperMix, Transgen®). The qRT-PCR reactions were manipulated by utilizing a Mastercycler®ep realplex real-time PCR system (ABI7500, MD, USA) with Bestar® SybrGreen qPCR mastermix (DBI, Bioscience Inc.,Germany) in a 20 μL reaction volume. The primers as shown in Table S2.

### Statistical analyses

Microsoft Office Excel 2016 was used to organize and process the data. Originpro 2016 was used to create the plots. SPSS statistical software installation package (version 11.0) was used to calculate the *p* value and tested for significant differences. The values are means ± standard deviation (SD) of the three independent experiments with at least three replicates for each. Differences among treatments were analyzed by one-way analysis of variance (ANOVA) integrated with Duncan’s multiple range test, with *P* < 0.05 as the threshold.

## Supplementary Information

**Additional file 1: Figure S1. **The expressions of the key *PAL*, *CHI* and *CHS* key genes in radish hypocotyls. **Table S1.**The expression levels of anthocyanin biosynthesis related structure genes. **Table S2.**The nucleotide sequence of primers used in the qRT-PCR.

## Data Availability

All data generated or analysed during this study are included in this published article [and its supplementary information files].

## Abbreviations

NO: Nitric oxide
NR: Nitrate reductase
NOS: Nitric oxide synthase
NOA1: Nitric oxide associated 1
EBGs: Early biosynthesis genes
PAL: Phenylalanine ammonia lyase
CHS: Chalcone synthase
CHI: Chalcone isomerase
F3H: Flavanone-3-hydroxylase
LBGs: Late biosynthesis genes
DFR: Dihydroflavonol 4-reductase
ANS: Anthocyanidin synthase
LDOX: Leucoanthocyanidin dioxygenase
UF3GT: UDPglucose flavonoid-3-O- glucosyltransferase
PAP1: Production of anthocyanin pigment1
PAP2: Production of anthocyanin pigment2
cPTIO: Carboxy-PTIO
ZNPP: ZincProtoporphyrin
SNP: Sodium Nitroprusside
NIA: (Nitrate reductase [NADH])
CO: Carbon monoxide
HO-1: Heme oxygenase-1
