# Supplementary material for: Improving the anthocyanin accumulation of hypocotyls in radish sprouts by hemin-induced NO
Source: BMC Plant Biol. 2022 Apr 30;22:224. doi: 10.1186/s12870-022-03605-w (PMC9055698; doi:10.1186/s12870-022-03605-w)
Supplement: Supplementary file 1 — Additional file 1: Figure S1. The expressions of the key PAL, CHI and CHS key genes in radish hypocotyls. Table S1.The expression levels of anthocyanin biosynthesis related structure genes. Table S2.The nucleotide sequence of primers used in the qRT-PCR. [file 12870_2022_3605_MOESM1_ESM.pptx]

## Slide 1
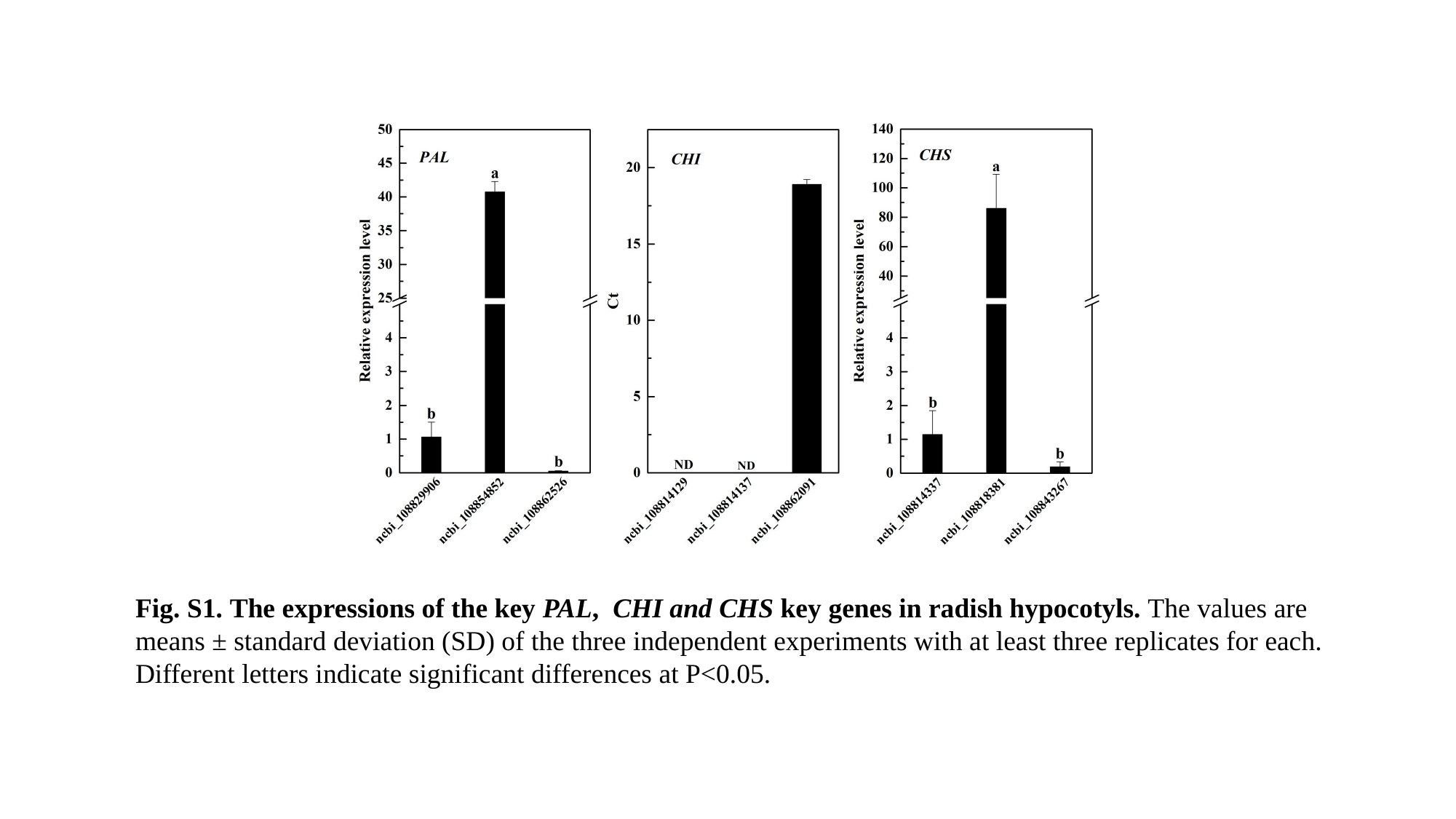

Fig. S1. The expressions of the key PAL, CHI and CHS key genes in radish hypocotyls. The values are means ± standard deviation (SD) of the three independent experiments with at least three replicates for each. Different letters indicate significant differences at P<0.05.

## Slide 2
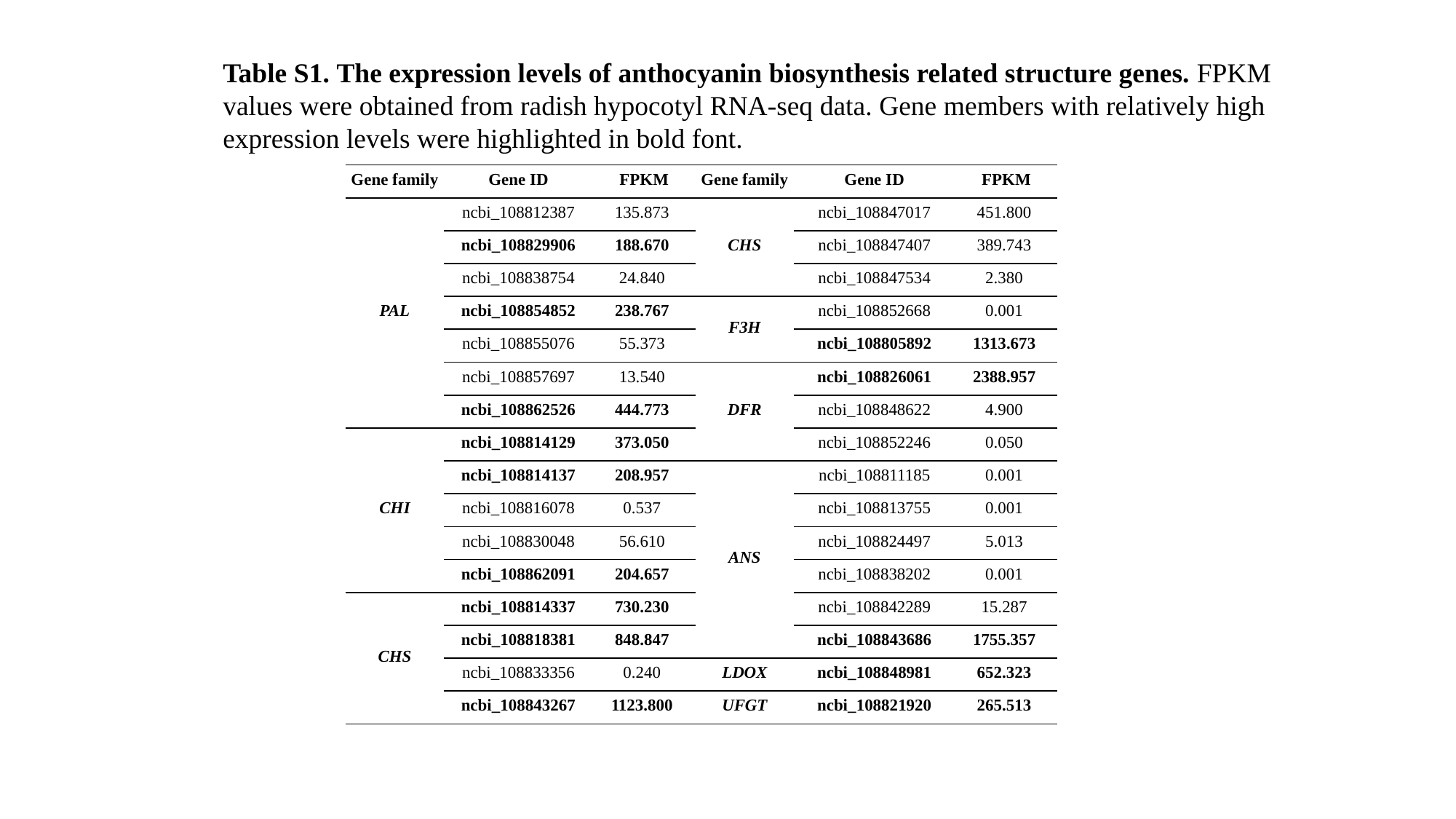

Table S1. The expression levels of anthocyanin biosynthesis related structure genes. FPKM values were obtained from radish hypocotyl RNA-seq data. Gene members with relatively high expression levels were highlighted in bold font.
| Gene family | Gene ID | FPKM | Gene family | Gene ID | FPKM |
| --- | --- | --- | --- | --- | --- |
| PAL | ncbi\_108812387 | 135.873 | CHS | ncbi\_108847017 | 451.800 |
| | ncbi\_108829906 | 188.670 | | ncbi\_108847407 | 389.743 |
| | ncbi\_108838754 | 24.840 | | ncbi\_108847534 | 2.380 |
| | ncbi\_108854852 | 238.767 | F3H | ncbi\_108852668 | 0.001 |
| | ncbi\_108855076 | 55.373 | | ncbi\_108805892 | 1313.673 |
| | ncbi\_108857697 | 13.540 | DFR | ncbi\_108826061 | 2388.957 |
| | ncbi\_108862526 | 444.773 | | ncbi\_108848622 | 4.900 |
| CHI | ncbi\_108814129 | 373.050 | | ncbi\_108852246 | 0.050 |
| | ncbi\_108814137 | 208.957 | ANS | ncbi\_108811185 | 0.001 |
| | ncbi\_108816078 | 0.537 | | ncbi\_108813755 | 0.001 |
| | ncbi\_108830048 | 56.610 | | ncbi\_108824497 | 5.013 |
| | ncbi\_108862091 | 204.657 | | ncbi\_108838202 | 0.001 |
| CHS | ncbi\_108814337 | 730.230 | | ncbi\_108842289 | 15.287 |
| | ncbi\_108818381 | 848.847 | | ncbi\_108843686 | 1755.357 |
| | ncbi\_108833356 | 0.240 | LDOX | ncbi\_108848981 | 652.323 |
| | ncbi\_108843267 | 1123.800 | UFGT | ncbi\_108821920 | 265.513 |

## Slide 3
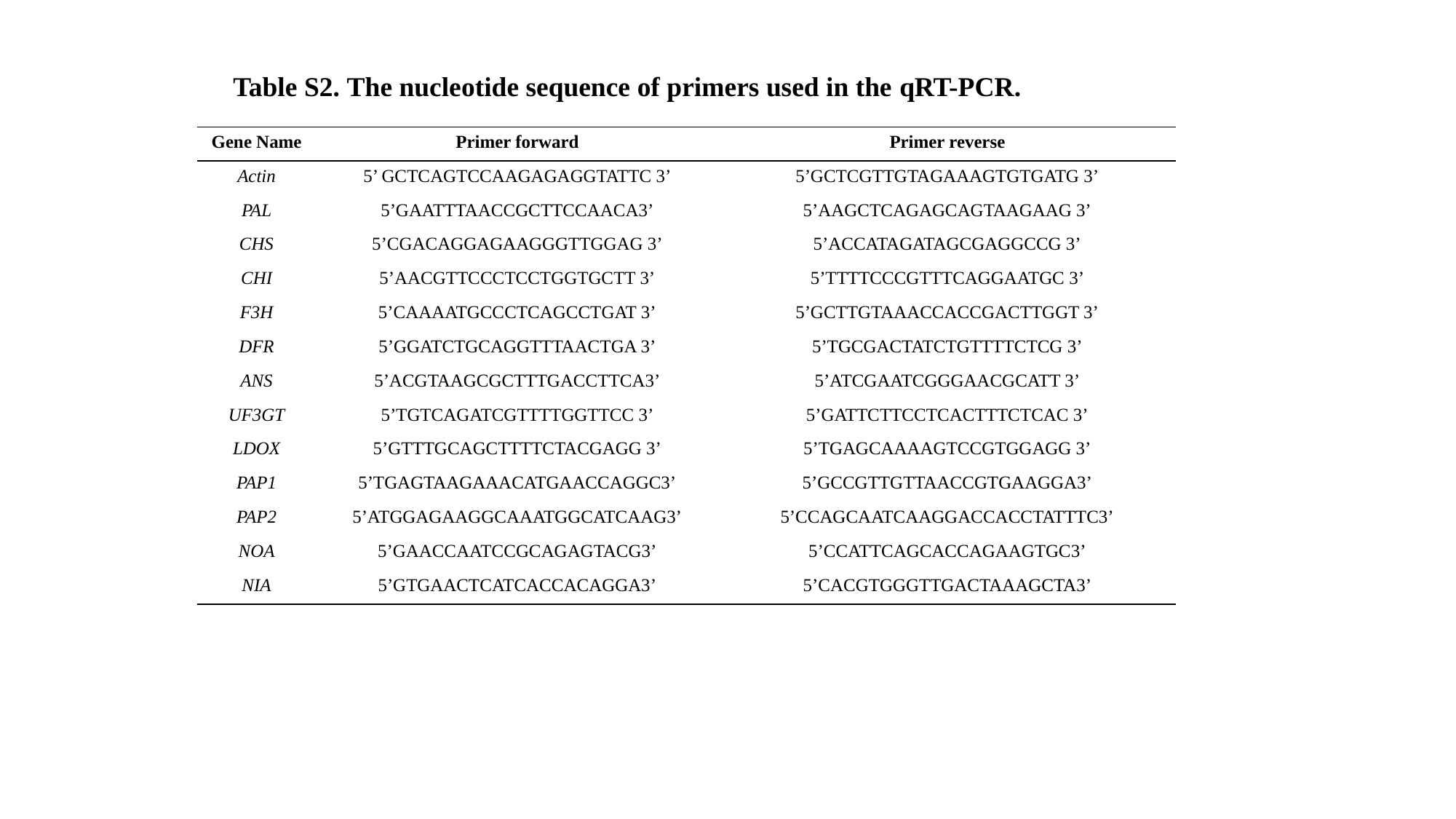

Table S2. The nucleotide sequence of primers used in the qRT-PCR.
| Gene Name | Primer forward | Primer reverse |
| --- | --- | --- |
| Actin | 5’ GCTCAGTCCAAGAGAGGTATTC 3’ | 5’GCTCGTTGTAGAAAGTGTGATG 3’ |
| PAL | 5’GAATTTAACCGCTTCCAACA3’ | 5’AAGCTCAGAGCAGTAAGAAG 3’ |
| CHS | 5’CGACAGGAGAAGGGTTGGAG 3’ | 5’ACCATAGATAGCGAGGCCG 3’ |
| CHI | 5’AACGTTCCCTCCTGGTGCTT 3’ | 5’TTTTCCCGTTTCAGGAATGC 3’ |
| F3H | 5’CAAAATGCCCTCAGCCTGAT 3’ | 5’GCTTGTAAACCACCGACTTGGT 3’ |
| DFR | 5’GGATCTGCAGGTTTAACTGA 3’ | 5’TGCGACTATCTGTTTTCTCG 3’ |
| ANS | 5’ACGTAAGCGCTTTGACCTTCA3’ | 5’ATCGAATCGGGAACGCATT 3’ |
| UF3GT | 5’TGTCAGATCGTTTTGGTTCC 3’ | 5’GATTCTTCCTCACTTTCTCAC 3’ |
| LDOX | 5’GTTTGCAGCTTTTCTACGAGG 3’ | 5’TGAGCAAAAGTCCGTGGAGG 3’ |
| PAP1 | 5’TGAGTAAGAAACATGAACCAGGC3’ | 5’GCCGTTGTTAACCGTGAAGGA3’ |
| PAP2 | 5’ATGGAGAAGGCAAATGGCATCAAG3’ | 5’CCAGCAATCAAGGACCACCTATTTC3’ |
| NOA | 5’GAACCAATCCGCAGAGTACG3’ | 5’CCATTCAGCACCAGAAGTGC3’ |
| NIA | 5’GTGAACTCATCACCACAGGA3’ | 5’CACGTGGGTTGACTAAAGCTA3’ |
